# Harnessing Medical Bioethics Mediation to Advance One Health Governance

**DOI:** 10.3390/vetsci13010008

**Published:** 2025-12-20

**Authors:** Olympia Lioupi, Polychronis Kostoulas, Gustavo Monti, Konstadina Griva, Charalambos Billinis, Costas Tsiamis

**Affiliations:** 1 Faculty of Public and One Health, School of Health Sciences, University of Thessaly, 43100 Karditsa, Greece; ktsiamis@uth.gr; 2 Infectious Disease Epidemiology (IDE), Wageningen University and Research (WUR), P.O. Box 9101, 6700 HB Wageningen, The Netherlands; gustavo.monti@wur.nl; 3 Lee Kong Chian School of Medicine, Nanyang Technological University, Singapore 639798, Singapore; konstadina.griva@ntu.edu.sg; 4 Faculty of Veterinary Medicine, School of Health Sciences, University of Thessaly, 43100 Karditsa, Greece; billinis@uth.gr

**Keywords:** one health governance, medical-bioethics mediation, conflict resolution, antimicrobial resistance, zoonotic disease control, environmental stewardship, participatory decision-making

## Abstract

One Health envisions integrated governance across human, animal, and environmental systems to prevent and respond to complex health threats. Despite its global endorsement, One Health implementation often falters due to institutional fragmentation, power asymmetries, and ethical tensions that erode trust and cooperation. This paper proposes the integration of medical-bioethics mediation within One Health governance as a structured, relational mechanism to manage conflict, foster ethical deliberation, and strengthen trust between sectors and communities. We develop a conceptual framework to apply the mediation principles of neutrality, confidentiality, respect, and shared problem-solving beyond clinical ethics toward multisectoral One Health contexts. The framework is illustrated through domain-specific examples from zoonotic disease control, antimicrobial resistance, and environmental health. Medical bioethics mediation can advance conflict transformation, ethical reflection, participatory decision-making, and policy alignment, thereby supporting transparent negotiation of values and institutionalized dialogue of different One Health actors. Future research should pilot mediation-based governance models and assess their effects on intersectoral trust, collaborative capacity, and integrated health outcomes.

## Simple Summary

Health threats such as infectious diseases, antibiotic resistance, and environmental changes involve people, animals, and nature together. Because they cross many areas of society, they often lead to disagreements between farmers, veterinarians, public health officials, environmental groups, and local communities. Such disagreements can slow down disease control, weaken cooperation, and reduce trust. This article explores how medical-bioethics mediation, a method used in hospitals to help doctors and families resolve conflicts, can also help different groups work together in broader health and environmental decisions. Mediation offers a respectful and neutral space where people can express concerns, understand each other’s viewpoints, and find solutions that everyone can support. Application of these skills to national planning, disease response, and environmental management, societies may reduce conflict, improve communication, and build trust across sectors. This approach can help create fairer, more effective, and more sustainable decisions that benefit both communities and the natural world.

## 1. Introduction

The One Health (OH) paradigm calls for coordinated and equitable governance across human health, veterinary medicine, wildlife management, agriculture and environmental sectors to sustainably prevent, detect and respond to threats at the human–animal–environment interface [1,2,3,4,5]. OH is now recognized as a unifying and transdisciplinary framework that mobilizes multiple sectors and communities to optimize the health of people, animals, and ecosystems through shared responsibility, communication, collaboration, and capacity building. It is both a practical coordination model and an ethical commitment to shared responsibility for the health of humans, animals, and ecosystems. Despite increasing adoption by governments and international agencies, implementation remains hindered by fragmented governance structures, inadequate and uneven resource allocation, and weak mechanisms for cross-sectoral coordination and accountability [6]. These barriers are evident in both low-income settings, where limited institutional capacity and funding shortfalls constrain sustained implementation [7] and high-income contexts, where overlapping mandates, siloed governance, and inconsistent financing frameworks continue to impede integration and long-term collaboration [8].

Recent global outbreaks, such as H5N1 avian influenza [9], Rift Valley Fever [10] and COVID-19 [11], have exposed persistent tensions between ministries, local actors, and communities regarding the design and enforcement of disease control measures, including compensation, vaccination, culling, and mobility restrictions [6,8]. Similarly, antimicrobial resistance (AMR) initiatives continue to struggle with harmonizing antibiotic use guidelines across human and animal sectors and reconciling public health imperatives with farmers’ livelihoods [12]. Comparable disputes also emerge in the environmental health domain, particularly over wildlife culling, habitat conservation, and industrial pollution, where competing interests often impede coherent policy responses [13]. These disagreements are often not technical but arise from differing values, priorities, and levels of authority, as well as persistent mistrust between sectors [4,6,8].

Medical bioethics mediation emerged within clinical ethics and healthcare conflict management as a structured, voluntary process that facilitates dialogue between parties in disagreement, helping them identify interests, clarify misunderstandings, and reach mutually acceptable resolutions without adjudication [14,15,16,17]. It emphasizes neutrality, confidentiality, respect, and active listening which are principles that enable moral repair and restore trust in strained relationships [18,19]. In clinical practice, mediation trains clinicians to reframe patient and family behaviour, explore multiple perspectives, and distinguish positions from underlying interests [15]. It is a voluntary, flexible, and confidential process that promotes open communication, empathy, and the preservation of relationships [20] where the mediators do not impose solutions and the parties may withdraw at any time. While traditionally applied at the bedside or within hospitals to manage disputes between clinicians, patients, and families, its core philosophy, which is based on structured dialogue, empathy, and shared problem-solving, offers broader relevance. We propose that these principles be extended beyond the clinical setting to guide multisectoral deliberations in OH and public health governance. Applied at the population level, medical-bioethics mediation can serve as a preventive tool that improves communication between ministries, professionals, and communities, reducing misunderstandings and polarization before crises escalate. The integration of medical bioethics mediation into OH governance could strengthen shared responsibility for health across sectors and improve coordination on complex problems.

This paper develops a conceptual framework for integrating medical bioethics mediation into OH governance and illustrates its relevance through examples from zoonotic disease control, AMR, and environmental health. The aim is to demonstrate how mediation’s core principles, neutrality, confidentiality, respect, and structured dialogue, can strengthen trust, ethical deliberation, and multisectoral cooperation within OH systems.

## 2. Theoretical Foundations

The theoretical approaches presented in this section are not intended as mutually exclusive alternatives, but they offer complementary perspectives and collectively inform the conceptual framework we develop in the following sections.

### 2.1. Medical Bioethics Mediation and the Dubler–Fisher Model

Medical bioethics mediation draws on clinical ethics practice to address conflict through structured dialogue, and its principles translate directly to the relational challenges of OH. Mediation emerged in the 1990s to resolve clinical ethics conflicts. The Dubler–Fisher model [14] emphasizes that mediation should foster respect, relationship repair and shared decision-making. The mediator, who is neutral, ensures that all parties are heard, and helps them reframe disputes as joint problems to be solved collaboratively. Mediation promotes open communication, empathy, confidentiality, and can preserve relationships and prevent lengthy litigation [21,22]. Clinicians can be taught to view conflicts from multiple perspectives and to distinguish positions from underlying interests to facilitate resolution [15]. We advocate that the skills of active listening, reframing and identifying shared values are directly transferable to intersectoral OH conflicts, where parties often misunderstand each other’s motivations. Thus, the Dubler–Fisher approach complements existing OH coordination platforms by helping to repair trust and ensuring that all parties are heard.

### 2.2. Deliberative Democracy

Deliberative democratic theory explains why OH governance requires spaces for reasoned, inclusive dialogue, that is, spaces that mediation can help structure and safeguard. Deliberative democracy theory supports mediated dialogue as a vehicle for reasoned consensus in pluralistic systems. A review of the literature on deliberative democracy and shared decision-making in healthcare, identified structural challenges such as professional hierarchies, informational asymmetries, and time constraints. Yet, at the same time promising developments have emerged like clearer policy frameworks, training in shared decision-making, and broader public engagement mechanisms [23]. Deliberative theory views democracy as a process of reasoned discussion between equal participants, not simply voting or aggregating preferences [24]. Such deliberative approaches have gained prominence, within healthcare and public health governance, as they enable informed, two-way interaction between decision makers and affected publics, promoting legitimacy, transparency, and social learning [25]. Inclusive deliberation aligns closely with the OH paradigm’s requirement for cross-sectoral negotiation and reflexivity. Medical bioethics mediation, as a structured deliberative process, can operationalize these democratic principles by ensuring balanced information exchange, equitable participation, and procedurally fair outcomes that will reflect collective reasoning rather than adversarial compromise. This distinctively relational function addresses gaps in existing OH dialogue structures, which often lack mechanisms to correct power imbalances or enable trust-building across sectors.

### 2.3. Relational Autonomy and Ethics of Care

Relational autonomy and care ethics highlight the interdependence at the core of OH systems and explain why a mediating process grounded in empathy, stewardship, and mutual responsibility is essential. Traditional bioethics emphasizes individual autonomy and negative liberty. Relational autonomy, rooted in the ethics of care, views autonomy as the ability to act and decide within relationships of mutual dependence. It demands engagement from professionals and institutions to support and co-shape the values of patients and communities. Empirical work in clinical ethics shows that such relational reasoning involves balancing respect for patient preferences with moral responsibility for vulnerable persons, acknowledging dignity, and exercising patience in shared deliberation [26]. Within OH, this perspective extends beyond human relationships: it situates decision-making within the interdependence of humans, animals, and ecosystems at the population level. Here, care becomes synonymous with stewardship, a shared moral responsibility for sustaining the conditions of collective well-being [27,28]. At the population level, medical bioethics mediation builds this ethic into governance by encouraging decisions that are made jointly, rather than imposed from above.

Together, these theoretical foundations frame medical bioethics mediation as both a normative and operational bridge between ethical principles and multisectoral action in OH governance. They show how technical coordination alone is insufficient, and why a relational approach is required to address mistrust, power asymmetries, and value conflict across sectors. With the reframe of conflict as an opportunity for learning and joint problem-solving, mediation strengthens the ethical resilience of OH systems and supports the development of governance structures that are more equitable and adaptive.

## 3. Rationale for Integrating Medical Bioethics Mediation into OH

### 3.1. Management of Diverse Conflicts

Multi-stakeholder OH initiatives frequently confront domain-specific conflicts that hinder cooperation. In zoonotic disease control, farmers, veterinarians, and public health authorities debate culling versus compensation or vaccination mandates. Such debates reflect deeper asymmetries in power, knowledge, and perceived risk and are compounded by institutional fragmentation and differing accountability frameworks across sectors [29]. These tensions often stem from inadequate mutual understanding and coordination between interdependent actors.

Participatory workshops in Burkina Faso’s anthrax surveillance initiative exemplify how inclusive dialogue can align epidemiological goals with livelihood priorities, converting adversarial debates into co-developed plans that reinforce trust and shared responsibility [30]. These experiences show the need for approaches that can turn conflict into constructive dialogue, a role for which medical bioethics mediation is well suited [31]. Other dialogue-based approaches within OH ethics, such as those proposed by Lerner [32] and by Foster and colleagues [33], also offer valuable frameworks for structuring ethical deliberation across sectors.

Comparable tensions underlie AMR governance, where health protection must be balanced with social and economic realities. Coordinating antibiotic stewardship across human, animal, and environmental sectors requires technical alignment, shared surveillance frameworks and equitable governance mechanisms, underscoring the need for integrated OH approaches [34]. Harmonization of antibiotic use guidelines across sectors depends on sustained negotiation, transparent data sharing, and consensus-building processes that align public health imperatives with agricultural and environmental priorities. Efforts in India to reconcile veterinary and clinical antibiotic policies highlight the importance of iterative cross-sectoral dialogue and institutional learning in achieving durable policy coherence [12].

Finally, environmental health conflicts, from wildlife management to land use and pollution, often expose tensions between scientific authorities and indigenous or local communities whose stewardship knowledge and relational worldviews remain undervalued within dominant governance frameworks. As shown in Australia, the documentation and application of indigenous biocultural knowledge have illuminated both the ethical and practical challenges of integrating traditional ecological insights into contemporary ecosystem governance [35]. Hence, there is a need for equitable bridging of knowledge systems, translation, negotiation, and synthesis, to cultivate mutual respect, trust, and co-produced knowledge [35,36]. Integration of indigenous and local knowledge into OH is an ethical imperative and a pragmatic pathway toward inclusive, adaptive, and resilient environmental stewardship.

These examples show that OH governance requires mechanisms that can mediate ethical disagreements and support shared accountability across sectors, particularly in situations where disputes transcend sectoral boundaries and generate cascading effects across human, animal, and ecosystem health. These are precisely the dimensions that medical bioethics mediation is uniquely designed to support.

### 3.2. Ethical and Governance Gaps

Managing these conflicts requires ethical frameworks that integrate human, animal, and environmental interests within a shared moral space. Principles such as autonomy, beneficence, non-maleficence, justice, solidarity, precaution, and intergenerational equity are fundamental to OH governance across zoonotic, environmental, and AMR contexts [37,38]. However, their operationalization across sectors remains inconsistent. Procedural values, such as transparency, deliberation, and participation, are seldom institutionalized, and affected communities are rarely co-designers of interventions [39]. Comparative assessments across Africa and Asia reveal that weak governance, insufficient collaboration, and poor communication between human, animal, and environmental health authorities continue to hinder OH implementation [37,40]. Hence the need for structured deliberation mechanisms capable of mediating conflicting values and rebuilding trust across sectors [41]. Thus, ethical governance in OH requires not only compliance with regulations but also continuous dialogue and cooperation to align the different interests involved.

### 3.3. Mediation as a Bridge

Transdisciplinary policy analysis emphasizes that identifying and understanding the often-conflicting interests, preferences, and values of multiple actors is a prerequisite for effective negotiation and collective action [42,43]. Analytical tools such as multicriteria decision analysis can structure stakeholder preferences but remain insufficient when dialogue and trust are absent. Transdisciplinary OH fosters co-leadership, iterative engagement, and conflict transformation across disciplinary and institutional boundaries [44].

Throughout this paper, our analysis is guided by a pluralistic ethical foundation that draws on widely recognized principles, such as autonomy, beneficence, non-maleficence, justice, solidarity, and stewardship, which underpin mediation practice and are consistent with principlism [45].

The ECDC [46] underscores that effective OH implementation depends on shared understanding of intersectoral linkages and the trust required for rapid, coordinated responses to complex zoonotic and environmental crises. Coordination failures often arise from insufficient institutionalized communication and weak relational accountability between sectors. These challenges show that OH governance is not only technical or administrative but also depends on building and maintaining strong relationships between sectors.

Medical bioethics mediation operationalizes these insights by providing a structured, interest-based process for dialogue and resolution that prioritizes respect, transparency, and collaboration [27,47]. Unlike existing participatory OH tools, like stakeholder consultations, risk communication platforms, or multisectoral working groups, mediation introduces a neutral third party, explicit confidentiality protections, and a formalized process for reframing disputes, thereby addressing power asymmetries and mistrust that technical coordination mechanisms often leave unresolved. In the context of OH governance, neutrality requires that the mediator has no decision-making authority, no institutional stake in the outcome, and no affiliation that could favour one party over another. Mediators are obliged to follow established professional standards that include impartial facilitation, confidentiality, and transparency about their role. As a deliberative mechanism, mediation complements technical instruments such as risk assessment and surveillance by addressing the interpersonal and value-based aspects of conflict that often undermine cooperation across sectors. Thus, mediation facilitates ethical reflection in practice and transforms stakeholder interactions from adversarial negotiation toward mutual understanding and shared problem solving.

Medical bioethics mediation functions primarily as a means to achieve ethical governance rather than a standalone governance model. Its value lies in embedding structured deliberation and relational repair within existing OH institutions rather than replacing them. This approach bridges the ethical and governance gaps identified earlier and transforms abstract commitments into tangible mechanisms for inclusive decision-making, trust repair, and the co-production of sustainable health futures.

## 4. Mechanisms of Action

### 4.1. Conflict Transformation

Conflict transformation does not just resolve disputes but changes the relationships and structures that generate them. Medical bioethics mediation shifts adversarial dynamics into collaborative problem-solving. In OH settings, mediators can help stakeholders articulate underlying concerns and values (e.g., livelihood, public health, animal welfare, cultural identity) rather than fixed positions (e.g., “no culling”) and reframe issues from zero-sum to shared interests (e.g., disease control benefits everyone). In this way, medical-bioethics mediation can transform distrust into mutual understanding and build durable partnerships. For instance, a recent participatory-planning workshop in Burkina Faso engaged human, animal and environmental health stakeholders to co-design an anthrax surveillance system, jointly define a shared vision, map inter-actor relations, and draft a consensual action plan. The entire process reportedly improved mutual understanding and generated trust across sectors [30]. Transdisciplinary policy frameworks likewise stress that understanding conflicting interests is a prerequisite to negotiation and collective action [2].

### 4.2. Ethical Deliberation Across Species

The OH ethical matrix encompasses human, animal and environmental ethics [48]. Medical bioethics mediation provides a space for ethical deliberation that integrates these plural values and ensures that the principles of beneficence, non-maleficence, justice and environmental responsibility are considered, and that stakeholders discuss trade-offs transparently. Relational autonomy encourages participants to acknowledge the vulnerability and interdependence of all beings [26]. The extension of the ethics of care to OH highlights the shared responsibility of humans, animals, and environmental sectors to protect the ecosystems they depend on. Through joint ethical deliberation, parties may accept precautionary measures or compensation schemes that balance human and animal interests [27]. Mediation also encourages consideration of long-term impacts and environmental responsibility, aspects often overlooked in technical risk assessments.

### 4.3. Participatory Decision-Making

OH interventions often suffer from top-down implementation while participatory decision-making that increases legitimacy, compliance and sustainability is often absent. The ECDC expert consultation emphasized that community engagement should follow a model that adapts to social and environmental contexts and builds mutual understanding [46]. In this context, medical bioethics mediation operationalizes participation by convening all affected parties (i.e., farmers, pastoralists, industry representatives, local leaders, and government agencies) within a structured, neutral process. Neutral facilitators ensure equitable speaking time, clarify scientific information and support joint drafting of agreements. This process embodies deliberative democracy’s requirements for balanced information and equal consideration of views [23]. A participatory design of OH surveillance systems, which was applied to AMR in Vietnam and Salmonella in France, involved a series of workshops in which stakeholders from human, animal and food-chain sectors jointly (i) defined the surveillance problem, (ii) mapped existing institutional relations, (iii) co-constructed a shared vision of a desired integrated surveillance system, and (iv) identified the actions needed to move from the current to the desired situation. This structured stakeholder dialogue fostered mutual understanding and joint ownership of surveillance goals [49]. Participatory and mediated dialogue can transform fragmented, sector-based governance into shared stewardship for collective health security, which is the interconnected well-being of humans, animals, ecosystems, and their shared social–ecological determinants at the population level.

### 4.4. Trust Building

Trust must be the cornerstone of effective OH surveillance, outbreak response and policy implementation. Systematic reviews identify ineffective collaboration, poor communication and lack of community engagement as major barriers to OH implementation [7]. The OH Joint Plan of Action notes that poor communication and lack of cooperation between stakeholders impede cross-sector work [37]. Participatory processes, transdisciplinary approaches and ECDC consultations all emphasize trust building. Medical bioethics mediation is a tool that fosters trust by ensuring confidentiality, respecting cultural differences and demonstrating impartiality [14]. It gives stakeholders voice and control and hence reduces perceptions of power imbalances and increases willingness to share data or comply with interventions. Trust developed through mediation also supports future collaboration by strengthening networks across sectors.

### 4.5. Policy Harmonization and Institutional Design

**Figure 1:**
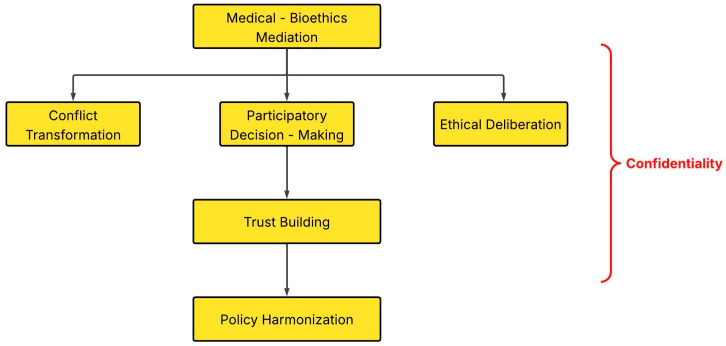
Mechanisms and pathways through which medical–bioethics mediation enhances OH governance.

Policy disharmony across sectors remains a major challenge in OH governance. Weak coordination mechanisms and unclear delineation of sectoral mandates further hinder implementation, especially in developing countries [7]. Experience from cross-sectoral policy platforms shows that negotiation and iterative dialogue are essential to reconcile conflicting priorities and regulatory cultures [27]. Medical bioethics mediation can facilitate such negotiations between ministries of health, agriculture, and environment to harmonize policies and align incentives. Transdisciplinary approaches emphasize co-leadership and the joint definition of objectives [2]. Medical bioethics mediation can also be formalized within national OH platforms or interministerial committees to support the drafting of memoranda of understanding, compensation schemes, or joint surveillance protocols. Incorporation of medical bioethics mediation within these structures helps make dialogue and shared accountability routine, shifting coordination from ad hoc responses to a more consistent collaborative approach. Ultimately, the legislative or procedural recognition of medical bioethics mediation demonstrates a commitment to fairness, transparency and long-term cooperation (Figure 1).

## 5. Potential Applications

### 5.1. Outbreak and Emergency Governance

During highly pathogenic avian influenza (H5N1) outbreaks, culling of poultry triggered intense disputes over compensation, livelihood loss, and food security. Structured mediation between veterinary authorities, public health agencies, poultry farmers, and local communities could have clarified scientific evidence, addressed economic concerns, and co-designed compensation mechanisms (Table 1). The ECDC consultation emphasized, cross-sectoral preparedness and mutual understanding of intersectoral linkages are essential for effective response [46]. Trained mediators within national rapid-response teams would enable real-time negotiation of quarantine measures, vaccination priorities, and resource allocation, while ensuring transparent risk communication and informed dialogue. Such mediators could be institutionally anchored by interfacing directly with existing national OH committees, Crisis Coordination Centres, and emergency operations platforms, to provide a neutral relational function that complements their technical and administrative mandates. This arrangement would allow mediation to be activated quickly during crises while at the same time remaining aligned with existing decision-making structures.

At the international level, the avian influenza experience also reveals how institutional frameworks shape cooperation and conflict. The emergence of the One World, One Health (OWOH) policy paradigm reflected efforts by the WHO, FAO and WOAH to reconcile overlapping mandates and reduce inter-agency tensions [50] through a shared cognitive framework that aimed to legitimize collaboration while at the same time maintain authority across sectors. This is essentially a process that mirrors the mediating function at the international scale. However, zoonotic crises inevitably expose conflicting narratives and value systems embedded in different disciplines and governance levels [51]. If OH is to remain effective, conflicts should be treated as opportunities for constructive dialogue rather than forced technical agreement. Medical bioethics mediation offers precisely this capacity to surface divergent perspectives, negotiate trade-offs transparently, and transform contested outbreaks into processes of shared learning and co-governance. Equally important, mediation can help address the moral injury and relational harm experienced by affected communities (i.e., farmers facing livelihood collapse, workers confronting stigma, or households subject to abrupt mobility restrictions) by ensuring that affected communities are heard and respected during crisis decision-making. In this way, mediation supports both practical decision-making and long-term resilience.

### 5.2. AMR and Health-System Integration

AMR is a global health crisis that threatens the effectiveness of treatment across human, animal, and environmental domains [34] and vividly illustrates the interdependence of these sectors. The excessive antimicrobial use in agriculture, livestock, and human medicine coupled with poor infection control, contaminated waste, and environmental dissemination has accelerated the spread of resistance [52]. The fight against AMR thus requires an integrated, multisectoral OH response. National AMR committees unite representatives from health, agriculture, environment, and finance ministries, yet divergent mandates and institutional cultures often lead to fragmented action. An Indian scoping review highlights the need for harmonized guidelines and convergence of policies across human and animal sectors [12]. Mediators can convene regular inter-ministerial dialogues to identify disagreements about responsibilities, economic costs, or trade effects, and help guide sectors toward joint policies on antibiotic use, surveillance, and enforcement.

Medical bioethics mediation could also extend beyond government to align pharmaceutical industries, veterinarians, and consumers on incentives for non-antibiotic alternatives, or facilitate farmer–regulator dialogues to co-design training and biosecurity measures suited to local realities. In India, widespread unregulated antimicrobial use by farmers, driven by limited knowledge and weak extension services, has been identified as a systemic gap [12]. Structured mediation in such contexts builds trust, connects behavioural change to practical incentives, and helps fragmented governance evolve into coordinated learning.

Ultimately, mediation could strengthen OH AMR governance as it helps sectors turn scientific evidence into shared commitments and sustained collaboration.

### 5.3. Environmental and Socio-Ecological Health

Environmental disputes often pit conservation priorities against community livelihoods and industrial interests. OH-oriented mediation offers a structured way to reconcile these competing claims through inclusive, evidence-informed dialogue. In cases of conflicts over pesticide use, vector control, water quality, or land conversion, mediators with ecological and health expertise can facilitate identification of co-benefits that simultaneously advance biodiversity protection and disease prevention.

The OH assessment of Ethiopia’s Chebera Churchura National Park illustrates these dynamics. Expansion, deforestation, and illegal logging have degraded habitats and intensified human–wildlife contact, increasing the risks of malaria, trypanosomiasis, and other neglected diseases in nearby communities [53]. Fragmented coordination between environment, health, and agriculture sectors underscored the need for multisectoral partnerships and governance reform, a process that medical-bioethics mediation is specifically designed to sustain. Facilitated dialogue between park authorities, communities, and public health agencies can co-design livelihood alternatives, equitable compensation, and joint surveillance programs. When grounded in ethical principles of respect, solidarity, and stewardship, such mediation transforms environmental conflict into collaboration. It links biodiversity conservation with public health goals and helps turn conflict into joint learning and planning that supports long-term resilience.

## 6. Illustrative Case Study: Mediated Response to Rift Valley Fever (Conceptual)

This case study is presented as a conceptual illustration, not an implemented intervention, and aims to demonstrate how medical-bioethics mediation could structure multisectoral deliberation during a Rift Valley Fever outbreak.

Rift Valley Fever (RVF) outbreaks in East Africa present complex challenges: human health authorities prioritize mosquito control to prevent human cases, veterinary authorities order livestock vaccination to protect animals and limit viral amplification, and pastoralists worry that vaccination and movement controls will lead to unpaid livestock deaths and market disruption [54,55]. In a mediated OH process, neutral facilitators trained in public health, veterinary science, and conflict resolution convene representatives from ministries of health, agriculture, and environment, pastoralist elders, women’s groups, and NGOs. The mediator begins by explaining the scientific evidence on RVF transmission and articulating the interests of each party. Pastoralists express concerns about compensation and trust while authorities explain regulatory mandates. Guided discussion reveals shared goals of preserving human lives, protecting livestock, and maintaining livelihoods and the parties jointly design a strategy that integrates vector control (e.g., larviciding, community education) with phased vaccination and establishes a transparent compensation mechanism, potentially backed by a regional contingency fund. Elders agree to support vaccination, ministries commit to timely payments, and all actors sign a memorandum of understanding. Follow-up meetings monitor progress and resolve emerging issues. Such a process illustrates how mediation can turn conflict into collaborative action.

## 7. Use of Generative AI Tools

During the preparation of this conceptual review, the authors used ChatGPT (GPT-5.1 model, OpenAI, OpenAI, San Francisco, CA, USA) to assist with language refinement and improvement of clarity. No part of the conceptual development, argumentation, interpretation, or theoretical framework was generated by the tool. All content produced with AI assistance was reviewed, edited, and verified by the authors, who take full responsibility for the accuracy and integrity of the manuscript.

## 8. Conclusions

The integration of medical-bioethics mediation into OH governance provides a concrete mechanism to translate ethical principles into operational practice. The greatest challenges of fragmented authority, mistrust, and value conflicts are not purely technical but relational and moral. Medical bioethics mediation addresses these dimensions directly by institutionalizing dialogue, transparency, and respect across human, animal, and environmental domains. Through facilitated engagement, disagreements become opportunities for ethical reflection, mutual learning, and shared stewardship. This approach fosters decisions that are legitimate, adaptive, and morally grounded, advancing OH from a general aspiration to a practical, shared approach to health protection.

Future research should design and test mediation-based models within existing OH coordination mechanisms to assess their effects on trust, collaboration, and equitable outcomes. Such work could strengthen preparedness and policy coherence while reaffirming the moral foundations of OH: dialogue, empathy, and shared care for the interdependent community of life.

## Figures and Tables

**Table 1:** Potential applications of medical–bioethics mediation across OH domains.

OH Domain	Conflict Dynamics	How Mediation Helps	Potential Benefits
Outbreak and Emergency Governance (e.g., H5N1, RVF)	Disputes over culling, compensation, and mobility restrictions Tensions between ministries, local authorities, and communities Distrust, misinformation, and fear of economic loss	Clarification of scientific evidence and regulatory mandates Neutral, real-time negotiation between veterinary, public health, and community actors Confidential space for expressing concerns Reframe of adversarial positions into shared goals	Faster agreement on containment strategies Improved acceptance of vaccination, movement controls, and compensation schemes Enhanced trust and transparent communication Strengthened crisis coordination and long-term resilience
AMR & Health System Integration	Divergent mandates between health, agriculture, environment sectors Resistance to harmonizing antibiotic use guidelines Farmer distrust, economic pressure, limited knowledge Poor coordination between public and private actors	Facilitation of inter-ministerial dialogue on stewardship, costs, and trade implications Alignment of veterinarians, farmers, and industry toward with shared incentives Support for co-design of training, surveillance, and enforcement strategies Build of trust needed for sustained behavior change	More coherent AMR policies across sectors Greater compliance with stewardship guidelines Reduced unregulated AMR use Stronger collaboration and adaptive learning across institutions
Environmental & Socio-Ecological Health	Livelihood vs. conservation tensions Disputes over land use, pollution, and pesticide control Marginalization of local knowledge Weak coordination between environment, health, and agriculture sectors	Creation of inclusive, evidence-informed dialogue between authorities, communities, and environmental groups Identification of co-benefits for biodiversity and disease prevention Facilitates of co-design of livelihood alternatives and compensation Supports for joint eco-health surveillance planning	Reduced conflict and increased cooperation Integrated biodiversity–health strategies Equity-centered environmental decision-making Strengthened ecological resilience and community trust

## Data Availability

Not applicable.

## Abbreviations

The following abbreviations are used in this manuscript:

AMR	Antimicrobial resistance
ECDC	European Centre for Disease Prevention and Control
FAO	Food and Agriculture Organization of the United Nations
H5N1	Highly pathogenic avian influenza A(H5N1) virus
NGO	Non-governmental organization
OH	One Health
OWOH	One World, One Health
RVF	Rift Valley Fever
WHO	World Health Organization
WOAH	World Organization for Animal Health
